# Supplementary material for: Confinement of unliganded EGFR by tetraspanin nanodomains gates EGFR ligand binding and signaling
Source: Nat Commun. 2023 May 9;14:2681. doi: 10.1038/s41467-023-38390-z (PMC10170156; doi:10.1038/s41467-023-38390-z)
Supplement: Supplementary file 1 — Supplementary Information [file 41467_2023_38390_MOESM1_ESM.pdf]

Sugiyama et al.

Confinement of unliganded EGFR by tetraspanin nanodomains gates EGFR ligand binding and signaling

Supplemental Information

Supplementary Tables 1-4
Supplementary Figures S1-S12

Supplementary Table 1. List of statistical tests used, p-values, and the number of independent experiments analyzed (ind. exp.) for data shown in Figure 1-8 and Supplementary Figure 1-12.

Figure panel	test	comparison	p-value	n (ind. exp.)
1C	two-way ANOVA, Tukey post-hoc test	Fab-Cy3B mobile class, 5 ng/mL vs 0 ng/mL	0.0002	5
		Fab-Cy3B mobile class, 10 ng/mL vs 0 ng/mL	0.0149	5
1E	two-way ANOVA, Tukey post-hoc test	SNAP (EGF-stim) vs EGF-Cy3B, immobile fraction	0.0338	3
		SNAP (EGF-stim) vs EGF-Cy3B, confined fraction	0.825	3
		SNAP (EGF-stim) vs EGF-Cy3B, mobile fraction	0.0878	3
2C	one-way ANOVA, Tukey post test	15 min EGF-stim vs 0 min EGF-stim	0.0354	3
2D	one-way ANOVA, Tukey post test	15 min EGF-stim vs 0 min EGF-stim	0.0323	4
2E	one-way ANOVA, Tukey post test	5 min EGF-stim vs 0 min EGF-stim	0.0379	10
		15 min EGF-stim vs 0 min EGF-stim	0.026	10
2F	Rank Sum Test	EGFR & CD81 coloc vs randomized position, 0 min EGF stim	<0.0001	3
	Rank Sum Test	EGFR & CD81 coloc vs randomized position, 5 min EGF stim	<0.0001	3
	Rank Sum Test	EGFR & CD81 coloc vs randomized position, 15 min EGF stim	<0.0001	3
2G	Rank Sum Test	EGFR & CD82 coloc vs randomized position, 0 min EGF stim	<0.0001	3
	Rank Sum Test	EGFR & CD82 coloc vs randomized position, 5 min EGF stim	<0.0001	3
	Rank Sum Test	EGFR & CD82 coloc vs randomized position, 15 min EGF stim	<0.0001	3
2H	Rank Sum Test	EGFR & CD151 coloc vs randomized position, 0 min EGF stim	<0.0001	3
	Rank Sum Test	EGFR & CD151 coloc vs randomized position, 5 min EGF stim	<0.0001	3
	Rank Sum Test	EGFR & CD151 coloc vs randomized position, 15 min EGF stim	<0.0001	3
3A	two-way ANOVA, Šídák's multiple comparisons test	Fab-Cy3B mobile class, control vs CD81 siRNA	<0.0001	5
		Fab-Cy3B confined class, control vs CD81 siRNA	0.0368	5

		Fab-Cy3B immobile class, control vs CD81 siRNA	0.017	5
3B	two-way ANOVA, Šídák's multiple comparisons test	Fab-Cy3B mobile class, control vs CD81 siRNA	0.002	5
3D	two-way ANOVA, Šídák's multiple comparisons test	Fab-Cy3B mobile class, control vs CD81 siRNA	0.0176	3
3E	two-way ANOVA, Šídák's multiple comparisons test	Fab-Cy3B mobile class, control vs CD81 siRNA	<0.0001	5
3F	two-way ANOVA, Šídák's multiple comparisons test	EGF-Cy3B mobile class, control vs CD81 siRNA	0.9644	5
3G	two-way ANOVA, Šídák's multiple comparisons test	Fab-Cy3B mobile class, control vs CD81 siRNA	0.0518	3
3H	two-way ANOVA, Šídák's multiple comparisons test	Fab-Cy3B confined class, control vs CD81 siRNA	0.0144	5
4A	one-way ANOVA, Tukey post test	Fab-Cy3B basal vs EGF-Cy3B 15s	0.0364	4
4B	one-way ANOVA, Tukey post test	control siRNA vs CD81 siRNA	0.0005	3
		control siRNA vs 4x tetraspanin siRNA	0.0128	3
4C	unpaired t-test, two tailed		0.0074	3
5B	two-way ANOVA, Šídák's multiple comparisons test	Fab-Cy3B mobile class, control vs clathrin siRNA	0.0059	4
5C	two-way ANOVA, Šídák's multiple comparisons test	EGF-Cy3B mobile class, control vs clathrin siRNA	0.1558	5
		EGF-Cy3B confined class, control vs clathrin siRNA	0.8308	5
		EGF-Cy3B immobile class, control vs clathrin siRNA	0.0301	5
5D	two-way ANOVA, Šídák's multiple comparisons test	Fab-Cy3B mobile class, control vs clathrin siRNA	0.0154	3
5E	two-way ANOVA, Šídák's multiple comparisons test	Fab-Cy3B mobile class, control vs clathrin siRNA	<0.0001	5
		Fab-Cy3B confined class, control vs clathrin siRNA	0.0007	5
5F	two-way ANOVA, Šídák's multiple comparisons test	EGF-Cy3B mobile class, control vs clathrin siRNA	0.0003	5
		EGF-Cy3B confined class, control vs clathrin siRNA	0.0254	5
		EGF-Cy3B immobile class, control vs clathrin siRNA	0.3457	5

5H	two-way ANOVA, Šídák's multiple comparisons test	Fab-Cy3B confined class, control vs clathrin siRNA	0.0125	5
5I	two-way ANOVA, Šídák's multiple comparisons test	EGF-Cy3B confined class, control vs clathrin siRNA	<0.0001	5
		EGF-Cy3B immobile class, control vs clathrin siRNA	0.046	5
5J	one-way ANOVA, tukey post test	15 min EGF stim vs 0 min EGF stim	0.0202	3
5K, left	Rank Sum Test	Clathrin & CD81 coloc vs randomized position clathrin channel, 0 min EGF stim	<0.0001	3
	Rank Sum Test	Clathrin & CD81 coloc vs randomized position clathrin channel, 5 min EGF stim	<0.0001	3
	Rank Sum Test	Clathrin & CD81 coloc vs randomized position clathrin channel, 15 min EGF stim	<0.0001	3
5K, middle	Rank Sum Test, Šídák's correction, larger of three comparisons	Clathrin & (CD81w/EGFR) coloc vs randomized position, 0 min EGF stim	0.0251	3
	Rank Sum Test, Šídák's correction, larger of three comparisons	Clathrin & (CD81w/EGFR) coloc vs randomized position, 5 min EGF stim	0.1851	3
	Rank Sum Test, Šídák's correction, larger of three comparisons	Clathrin & (CD81w/EGFR) coloc vs randomized position, 15 min EGF stim	<0.0001	3
5K, right	Rank Sum Test, Šídák's correction, larger of three comparisons	Clathrin & (CD81w/oEGFR) coloc vs randomized position, 0 min EGF stim	0.0594	3
	Rank Sum Test, Šídák's correction, larger of three comparisons	Clathrin & (CD81w/oEGFR) coloc vs randomized position, 5 min EGF stim	0.5493	3
	Rank Sum Test, Šídák's correction, larger of three comparisons	Clathrin & (CD81w/oEGFR) coloc vs randomized position, 15 min EGF stim	0.0224	3
7C	two-way ANOVA, Šídák's multiple comparisons test	Fab-Cy3B mobile class, 10 ng/mL vs 100 ng/mL EGF stim.	0.0252	3
		Fab-Cy3B confined class, 10 ng/mL vs 100 ng/mL EGF stim.	0.8619	3
		Fab-Cy3B immobile class, 10 ng/mL vs 100 ng/mL EGF stim	0.039	3
7F	two-way ANOVA, Šídák's multiple comparisons test	control siRNA vs CD81 siRNA, 1 min EGF stim	0.0198	4
8A	unpaired t-test, two tailed		0.0394	6

8B	two-way ANOVA, Šídák's multiple comparisons test	EGF-Cy3B mobile class, control vs erlotinib	<0.0001	3
		EGF-Cy3B confined class, control vs erlotinib	0.0141	3
		EGF-Cy3B immobile class, control vs erlotinib	0.0296	3
8G	two-way ANOVA, Šídák's multiple comparisons test	EGF-Cy3B confined class, control vs erlotinib	0.0031	3
		EGF-Cy3B mobile class, control vs erlotinib	0.0008	3
8J	two-way ANOVA, Šídák's multiple comparisons test	EGF-Cy3B confined class, control vs erlotinib	0.001	3
S1E	two-way ANOVA, Šídák's multiple comparisons test	Fab-Cy3B mobile class, 100 ng/mL EGF vs basal	0.009	4
		Fab-Cy3B mobile class, 200 ng/mL EGF vs basal	<0.0001	4
S3J	two-way ANOVA, Šídák's multiple comparisons test	CD81 low (real vs randomized)	0.0401	3
		CD81 high (real vs randomized)	0.0037	3
S5A	two-way ANOVA, Šídák's multiple comparisons test	Fab-Cy3B mobile class, control vs CD81 siRNA	0.0089	3
S5B	two-way ANOVA, Šídák's multiple comparisons test	Fab-Cy3B mobile class, control vs CD81 siRNA	0.0023	3
		Fab-Cy3B immobile class, control vs CD81 siRNA	0.0253	3
S5C	two-way ANOVA, Šídák's multiple comparisons test	EGF-Cy3B mobile class, control vs CD81 siRNA	0.2309	3
		EGF-Cy3B confined class, control vs CD81 siRNA	0.8948	3
		EGF-Cy3B immobile class, control vs CD81 siRNA	0.3416	3
S5D	two-way ANOVA, Šídák's multiple comparisons test	Fab-Cy3B mobile class, control vs CD81 siRNA	0.0422	3
S5E	two-way ANOVA, Šídák's multiple comparisons test	Fab-Cy3B mobile class, control vs CD81 siRNA	0.0147	3
S5G	two-way ANOVA, Šídák's multiple comparisons test	Fab-Cy3B confined class, control vs CD81 siRNA	0.0722	3
S5H	two-way ANOVA, Šídák's multiple comparisons test	Fab-Cy3B confined class, control vs CD81 siRNA	0.0888	3
S6D	two-way ANOVA, Šídák's multiple comparisons test	Fab-Cy3B mobile class, control vs CD82 siRNA	0.0031	5

S6E	two-way ANOVA, Šídák's multiple comparisons test	Fab-Cy3B mobile class, control vs CD82 siRNA	0.0008	5
S6F	two-way ANOVA, Šídák's multiple comparisons test	EGF-Cy3B mobile class, control vs CD82 siRNA	0.0016	5
		EGF-Cy3B confined class, control vs CD82 siRNA	0.0376	5
		EGF-Cy3B immobile class, control vs CD82 siRNA	0.5887	5
S6G	two-way ANOVA, Šídák's multiple comparisons test	Fab-Cy3Bconfined class, control vs CD82 siRNA	0.0042	3
S6H	two-way ANOVA, Šídák's multiple comparisons test	all comparisons	n.s.	3
S6I	two-way ANOVA, Šídák's multiple comparisons test	EGF-Cy3B confined class, control vs CD82 siRNA	0.0121	5
S7D	two-way ANOVA, Šídák's multiple comparisons test	Fab-Cy3B mobile class, control vs CD9 siRNA	0.0494	3
S7E	two-way ANOVA, Šídák's multiple comparisons test	Fab-Cy3B mobile class, control vs CD9 siRNA	0.0012	5
S7F	two-way ANOVA, Šídák's multiple comparisons test	EGF-Cy3B mobile class, control vs CD9 siRNA	0.0158	5
S8D	two-way ANOVA, Šídák's multiple comparisons test	Fab-Cy3B mobile class, control vs CD9 siRNA	0.004	3
S8E	two-way ANOVA, Šídák's multiple comparisons test	Fab-Cy3B mobile class, control vs CD9 siRNA	0.0008	5
S8F	two-way ANOVA, Šídák's multiple comparisons test	EGF-Cy3B mobile class, control vs CD9 siRNA	0.0016	3
		EGF-Cy3B confined class, control vs CD9 siRNA	0.0376	3
S8G	two-way ANOVA, Šídák's multiple comparisons test	Fab-Cy3B confined class, control vs CD9 siRNA	0.0042	3
S8I	two-way ANOVA, Šídák's multiple comparisons test	EGF-Cy3B confined class, control vs CD9 siRNA	0.0118	5
S11C	two-way ANOVA, Šídák's multiple comparisons test	EGF-Cy3B, immobile class control vs afatinib	0.0333	4
		EGF-Cy3B, confined class control vs afatinib	0.009	4
		EGF-Cy3B, mobile class control vs afatinib	<0.0001	4
S11F	two-way ANOVA, Šídák's multiple comparisons test	EGF-Cy3B, confined class control vs afatinib	0.0353	4
		EGF-Cy3B, mobile class control vs afatinib	0.0002	4

S11I	two-way ANOVA, Šídák's multiple comparisons test	EGF-Cy3B, immobile class control vs gefinitib	0.0002	4
		EGF-Cy3B, confined class control vs gefitinib	0.0006	4
		EGF-Cy3B, mobile class control vs gefitinib	<0.0001	4
S12A	two-way ANOVA, Šídák's multiple comparisons test	EGF-Cy3B, immobile class control vs erlotinib	<0.0001	5
		EGF-Cy3B, confined class control vs erlotinib	0.0022	5
		EGF-Cy3B, mobile class control vs erlotinib	<0.0001	5
S12D	two-way ANOVA, Šídák's multiple comparisons test	EGF-Cy3B, immobile class control vs erlotinib	<0.0001	3
		EGF-Cy3B, confined class control vs erlotinib	<0.0001	3
		EGF-Cy3B, mobile class control vs erlotinib	<0.0001	3

Supplementary Table 2. List of primary antibodies for immunofluorescence staining and immunoblotting.

Name	Clone	Company	Cat. No.	IF dilution	IB dilution
CD81	1.3.3.22	Invitrogen	MA5-13548	1:100	N/A
CD82	C33	Cedarlane	CLX476AP	1:100	N/A
CD151	11G5a	Abcam	Ab33315	1:100	N/A
Clathrin Heavy Chain	D3C6	Cell Signaling Technology	4796	1:100	N/A
Caveolin-1	D46G3	Cell Signaling Technology	3267	1:100	N/A
Flotillin-1	D2V7	Cell Signaling Technology	18634	1:100	N/A
EGFR pY1068	Polyclonal	Invitrogen	44-788G	N/A	1:1000
EGFR pY1173	53A5	Cell Signaling	4407	N/A	1:1000
EGFR pY845	Polyclonal	Cell Signaling Technology	2231	N/A	1:1000
EGFR pY992	EM-12	Abcam	ab81440	N/A	1:1000
EGFR	Polyclonal	Santa Cruz Biotechnology	sc-03-G	N/A	1:1000
EGFR	mAb-108	N/A	N/A	50 ng/ml	N/A
EGFR	mAb-806	N/A	N/A	1 μg/ml	N/A
pAkt (S473)	D9E	Cell Signaling Technology	4060	N/A	1:1000
Akt	D0D4	Cell Signaling Technology	2920	N/A	1:1000
p-PLCγ	Polyclonal	Cell Signaling Technology	2821	N/A	1:1000
pERK	D13.14.4E	Cell Signaling Technology	4370	N/A	1:1000
ERK	137F5	Cell Signaling Technology	4695S	N/A	1:1000
GAPDH	14C10	Cell Signaling Technology	2118S	N/A	1:1000
Actin	D18C11	Cell Signaling Technology	8456S	N/A	1:1000

Supplementary Table 3. List of secondary antibodies for immunofluorescence and immunoblotting

Name	Company	Cat. No.	IF dilution	IB dilution
Anti-Goat HRP	Promega	V8051	N/A	1:20,000
Anti-Mouse HRP	Cell Signaling Technology	7076S	N/A	1:10,000
Anti-Rabbit HRP	Cell Signaling Technology	7074S	N/A	1:10,000
Anti-Rabbit AF488	Jackson ImmunoResearch	711-545-152	1:1000	N/A
Anti-Mouse Cy3	Jackson ImmunoResearch	715-165-150	1:1000	N/A
Anti-Mouse AF647 (Fcγ-specific)	Jackson ImmunoResearch	115-605-164	1:1000	N/A
Dylight 405 Anti-Rabbit	Jackson ImmunoResearch	111-405-003	1:1000	N/A

Supplementary Table 4. List of other reagents and catalog numbers used in this study.

Name	Company	Cat. No.
Human EGF Recombinant Protein	Thermo Fisher Scientific	PHG0313
Phorbol 12-myristate 13-acetate (PMA), PKC activator	Abcam	16561-29-8
Erlotinib	MedChemExpress	CP-358774
Gefitinib	MedChemExpress	ZD1839
Lapatinib	MedChemExpress	GW572016
Lipofectamine™ RNAiMAX Transfection Reagent	Invitrogen	13778075
DAPI	Roche	10236276001
Prolong™ Diamond Antifade Mountant with DAPI	Invitrogen	P36962
Cy3b NHS Ester	Cytiva Life Sciences	PA63100
Penicillin-Streptomycin	Life Technologies	15070063
DMEM/F-12 (1X)	Life Technologies	11330032
DMEM/F-12 (1X), no phenol red	Thermo Fisher Scientific	11039021
Ham's F-12 Nutrient Mix	Life Technologies	11765062
DMEM High Glucose	Life Technologies	11996065
DMEM High Glucose, no phenol red	Thermo Fisher Scientific	21063029
HEPES	Life Technologies	15630080
Insulin, Human	Roche	11376497001
Hydrocortisone	AK Scientific	C481
Fetal Bovine Serum, Qualified	Life Technologies	12483020
Doxycycline Hydrochloride	Bio Basic	24390-14-5
Puromycin Hydrochloride	Sigma Aldrich	P7255
SNAP-Surface® 488	New England BioLabs	S9124S
Paraformaldehyde 16% Aqueous Solution	Electron Microscopy Sciences	15710
SuperBlock™ Blocking Buffer	Thermo Fisher Scientific	37515
Bromophenol Blue	BioShop	BRO777.5
Protease Inhibitor Cocktail (100X)	BioShop	PIC001.1
Phosphatase Inhibitor Cocktail I	BioShop	PIC008.1
Sodium Orthovanadate	BioShop	SOV664.50
Okadaic Acid	Cell Signaling Technology	5934S

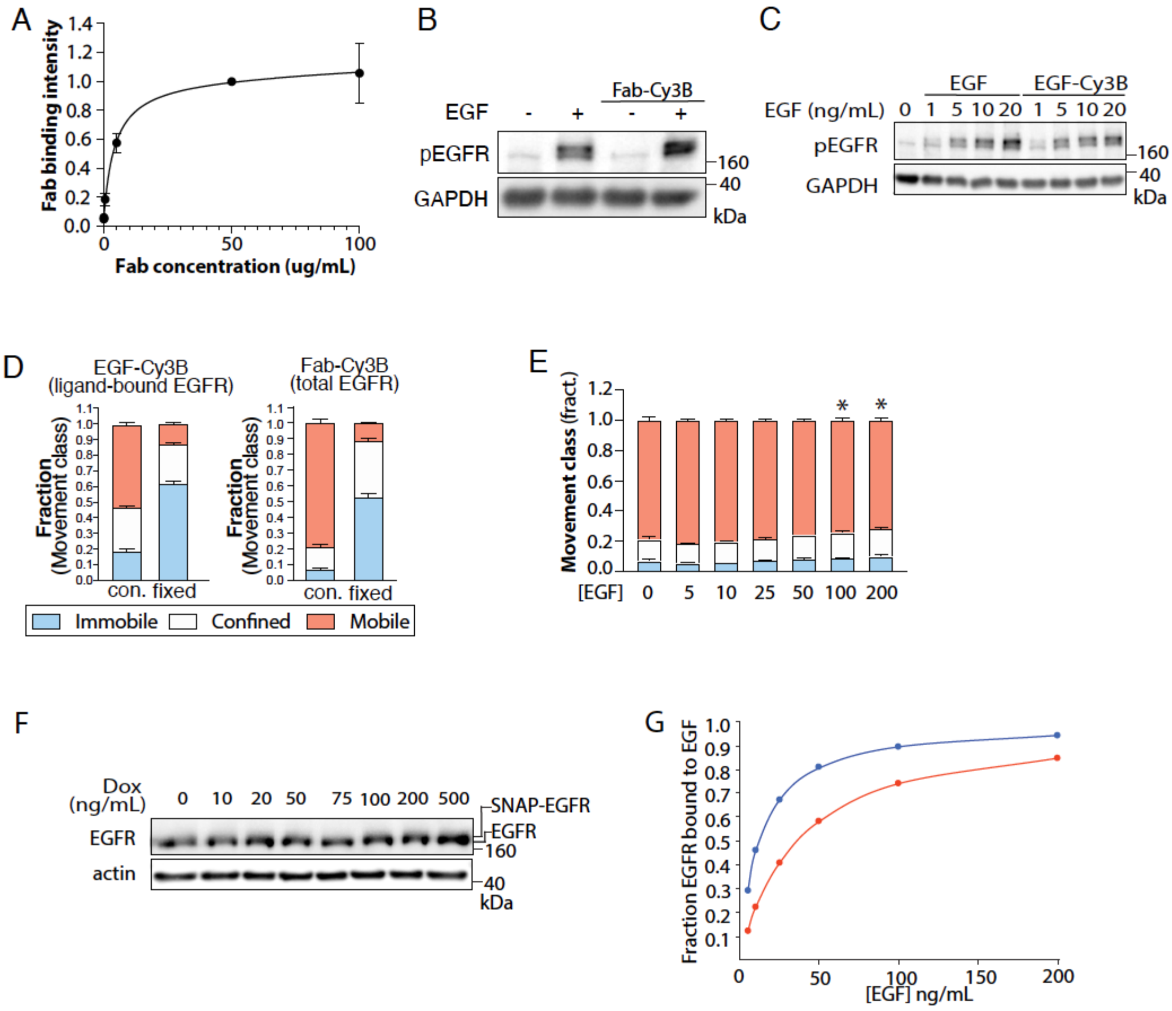

Supplementary Figure 1. Validation of labeling and tracking strategies used for single-particle tracking. (A) Intact ARPE-19 cells were treated with Fab-Cy3B at various concentrations, followed by fixation, imaging using widefield epifluorescence microscopy, and measurement of cell-associated Fab-Cy3B; shown are the means ± SE. (B) ARPE-19 cells were treated with Fab-Cy3B under saturating conditions (50 µg/mL), or left untreated, prior to labeling for 2 min with Cy3B-EGF. Shown are representative images as well as the quantification of cell surface EGF-Cy3B from *n*=4 independent experiments, showing the overall mean (bar) ± SE, and mean values from independent experiments (dots). Scale = 20 µm (C) ARPE-19 cells were stimulated with indicated concentrations of EGF-Cy3B or unlabelled EGF for 5 min. Shown are immunoblots of whole-cell lysates detecting phosphorylated EGFR (pY1068) or GAPDH (loading control). (D) ARPE-19 cells were subjected to methanol fixation (fixed) or not (control, con.), followed by labeling with either EGF-Cy3B or Fab-Cy3B. Results of SPT analysis showing the fraction of all EGFR tracks, as labelled by Fab-Cy3B (left panels), or showing the fraction of only ligand-bound EGFR, as labeling EGF-Cy3B (right panels) that exhibit mobile, confined or immobile behaviour. (E) Extended results of SPT analysis as per Fig. 1. Shown are the mean ± SE of the fraction of all EGFR tracks, as labelled by Fab- that exhibit mobile, confined or immobile behaviour. *, $p < 0.05$ compared to control (non-ligand stimulated) condition. (F) ARPE19 stable cells engineered for inducible expression of N-SNAP-EGFR were treated with doxycycline as indicated for 96h. Shown is a representative immunoblot of whole cell lysates showing detection of EGFR or actin. The N-SNAP-EGFR is ~20 kDa larger than the endogenous EGFR, but under the western blotting conditions examined here, both N-SNAP-EGFR and endogenous EGFR exhibit similar migration. N-SNAP-EGFR is expressed at

2-4x endogenous EGFR upon doxycycline induction in this range, ensuring non-perturbing conditions. (G) Calculation of the fraction of EGFR bound to EGF at different concentrations based on a K_d of 2 nM (red line) or 6 nM (blue line). This is based on Michaelis-Menten equation, which provides that Fraction bound = $(B_{max})[L]/([L]+K_d)$, and the assumption that B_{max} = 1. For panels showing western blots, approximate molecular weight is shown in kD. Statistical analysis and p-values are indicated in Supplementary Table 1. Source data are provided as a Source Data file.

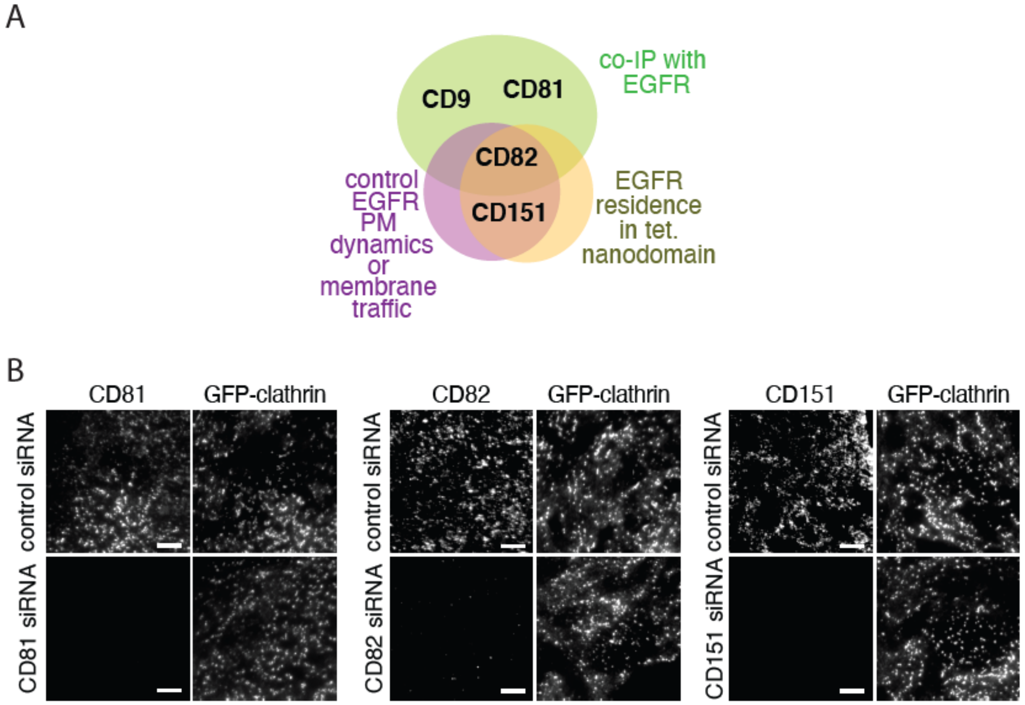

Supplementary Figure 2. Tetraspanin interactions with EGFR and tetraspanin antibody validation. (A) Diagram showing the previously published interactions (physical or functional) of 4 tetraspanins (CD9, CD81, CD82 and CD151) with EGFR. (B) Antibody validation for detection of CD151 or CD82 via TIRF microscopy. ARPE-19 cells stably expressing GFP-clathrin were treated with siRNA targeting CD81, CD82 or CD151, followed by labeling with either anti-CD81, anti-CD82 or -CD151 antibodies, as indicated. Shown are representative images obtained by TIRF-M. This figure shows images representative of two independent experiments. Scale 5 μm.

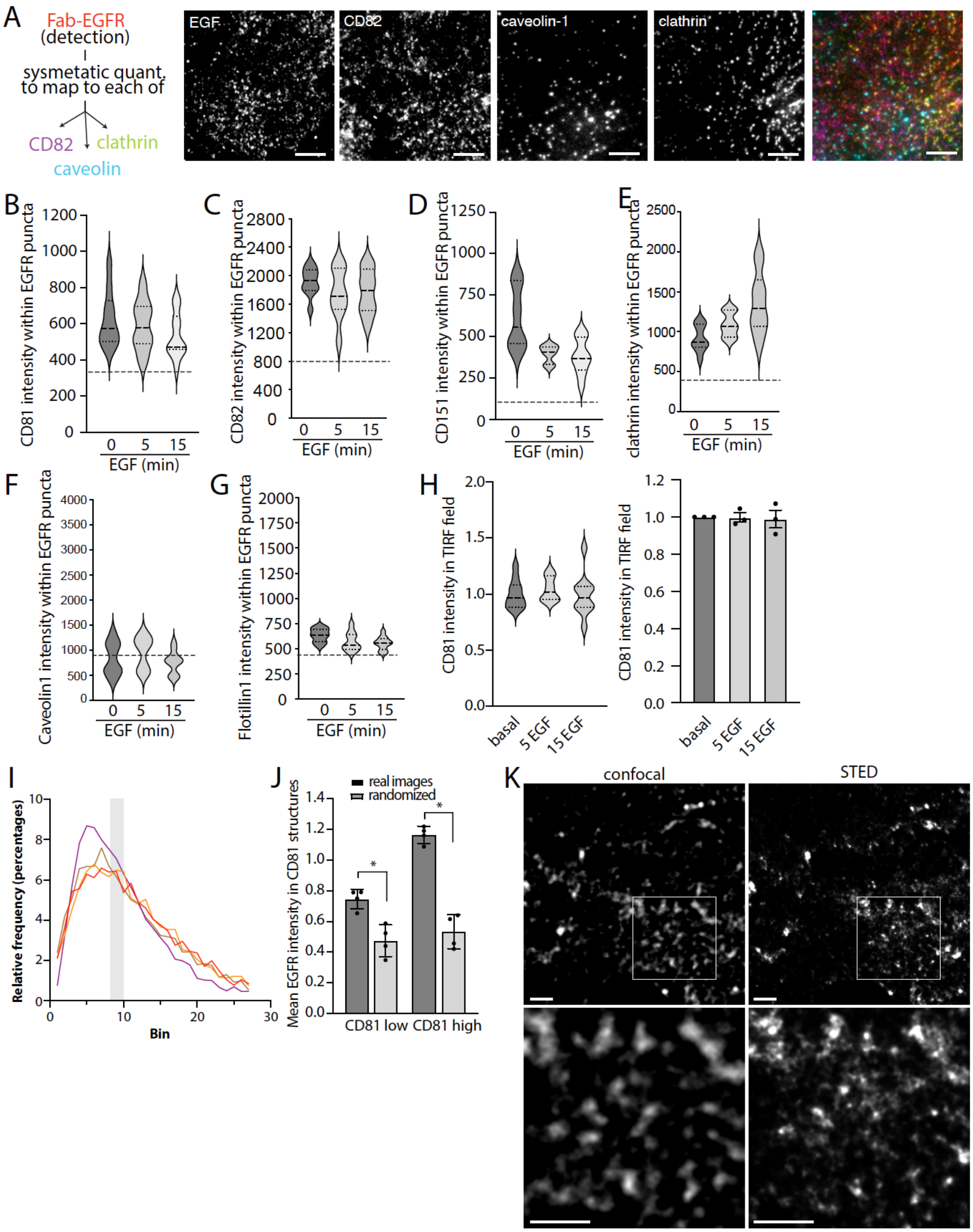

Supplementary Figure 3. Labeling of multiple nanodomain markers alongside EGFR. (A) Multi-channel parallel labeling of EGFR with multiple nanodomain markers. Diagram showing labeling strategy (left panels), which involves labeling of cells with Fab-Cy3B (to label total EGFR), followed by fixation and staining nanodomain markers. Shown are representative images obtained by TIRF-M of ARPE-19 stably expressing eGFP-clathrin labeled by IF staining for CD82 and caveolin-1, followed by labeling with Cy3B-Fab (to detect total EGFR). This figure shows images from two independent experiments. (B-G) Shown are results of detection of EGFR objects followed by *intensity-based analysis* of EGFR object

overlap with the indicated secondary channel (tetraspanins, clathrin, flotillin1, caveolin1) (as described in Methods). Also shown is the background overlap (horizonal dashed line) determined by repeating measurements of EGFR overlap with each secondary marker following rotation of one image by 180 degrees to randomize the marker overlap. Results are shown as the distribution of measurements in individual cells (violin plot), featuring the 25th, 50th and 75th percentiles (horizonal dashed lines). (H) The total levels of CD81 at the cell surface were determined by measuring the intensity of CD81 in TIRF images from Fig. 2. Shown are the levels of CD81 cell surface intensity in individual cells of a single experiment (left panels) as well as the mean (bar) of three (3) independent experiments (dots) ± SE; *, p <0.05 (right panel). (I-J) Images in Fig. 2 were further subjected to analysis to detect CD81 diffraction-limited objects using a similar method as the detection of EGFR objects shown in Fig. 2. (I) Shown is the intensity distribution of CD81 objects in these images from the 5th percentile to the 95th percentile of CD81 intensity in each experiment. Independent experiments are shown in different colors, each experiment involved analysis of >10 cells and >4500 CD81 objects. The vertical grey bar indicates the arbitrary threshold used to classify CD81 objects based on size for the subsequent figure. (J) Following CD81 detection, CD81 objects were classified as CD81-low (below the CD81 intensity median in that experiment) or CD81-high. Shown is the mean EGFR intensity (detected via Fab-Cy3B labeling) in CD81 objects (dots) ± SE; *, p <0.05. This analysis was performed in actual image pairs (“real images”) as well following randomization of the EGFR image (180-degree rotation); the latter allows measurement of background overlap of CD81 and EGFR signals. These experiments show that EGFR is significantly detected above background in both CD81-low and CD81-high objects, consistent with EGFR association with small-scale and large-scale CD81 assemblies. (K) STED microscopy following antibody labeling of endogenous CD81. Shown are representative micrographs of the same region of interest (ROI) obtained by confocal or STED, showing larger ROI (top panels) and magnified box (bottom panels). In the STED images, Scale = 5 μm. CD81 can be readily seen in both large and small objects, consistent with CD81 being present in both small- and large-scale assemblies. This figure shows images from one independent experiment. Statistical analysis and p-values are indicated in Supplementary Table 1. Source data are provided as a Source Data file.

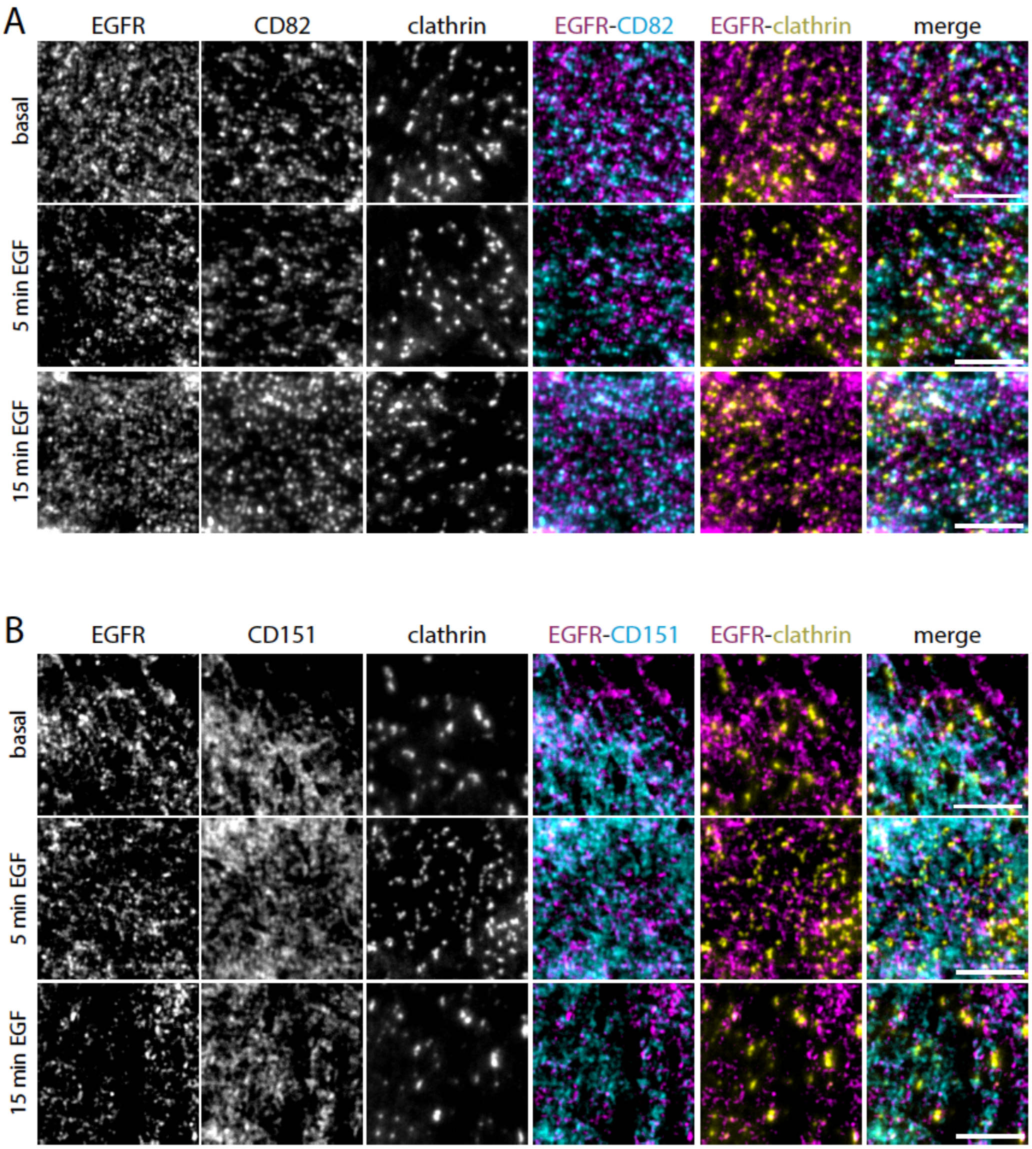

Supplementary Figure 4. Localization of EGFR relative to CD82, CD151 and clathrin at the cell surface. ARPE-19 cells stably expressing eGFP-clathrin were labeled with Fab-Cy3B (to label total EGFR) and stimulated with EGF as indicated, followed by fixation and staining with CD82 (A) or CD151 (B) antibodies. Shown for each are images obtained by TIRF-M representative of 3 independent experiments; antibody labeling of tetraspanins is highly specific (Supplementary Figure 2); similar experiments with labeling of CD81 were performed (Fig. 2). Scale = 5 μm.

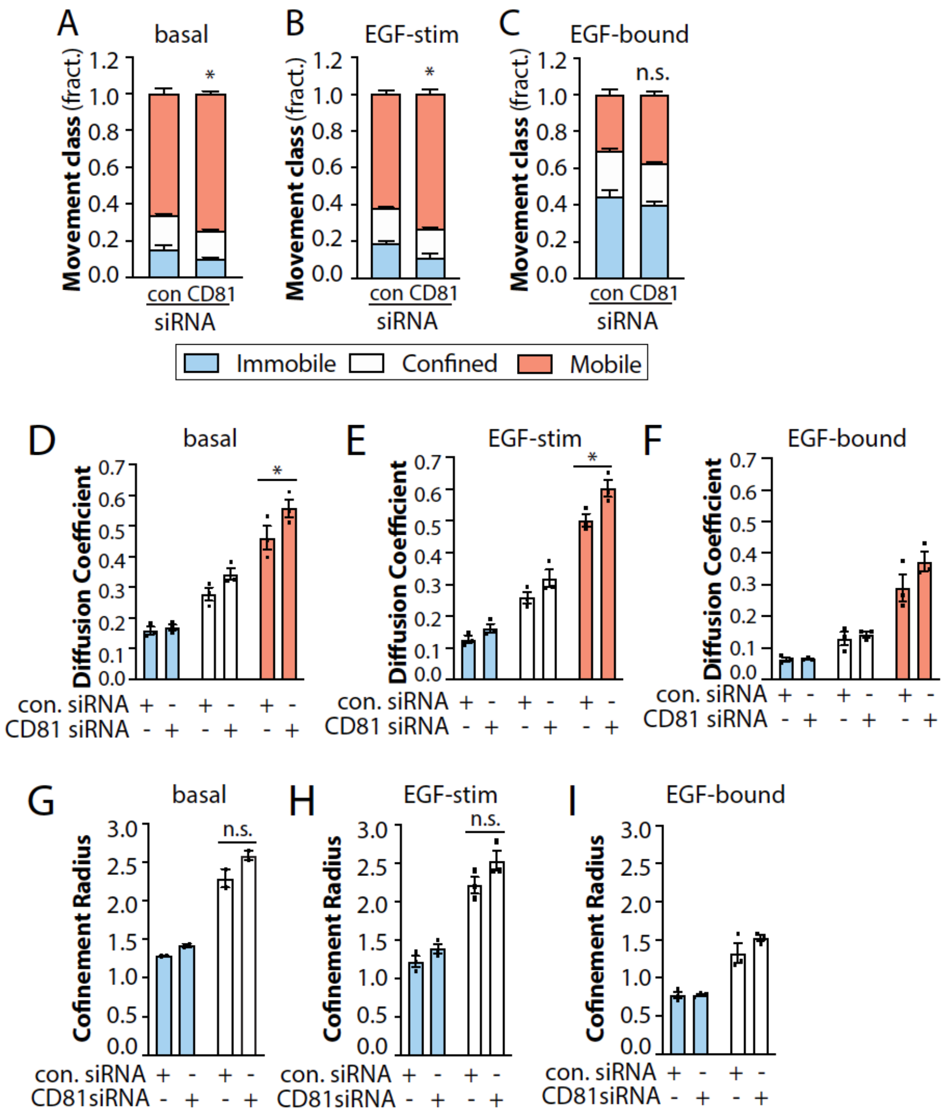

Supplementary Figure 5. Silencing CD81 impacts the diffusion coefficient of mobile EGFR in MDA-MB-231 cells. MDA-MB-231 cells were treated with siRNA to silence CD81 or non-targeting siRNA (control), as indicated. (A-I) Results of SPT analysis. The cells were then subjected to SPT using either Fab-Cy3B to label total EGFR in the absence (A, D, G) or presence (B, E, H) of unlabelled EGF, or labelled using EGF-Cy3B (C, F, I), to label only ligand-bound EGFR). Shown in A-C are the mean ± SE of the fraction of EGFR tracks in each mobility category (immobile, confined, mobile) under each condition, as well as in A-B the same data re-plotted to view the immobile and confined fractions. Also shown are mean ± SE of diffusion coefficient (D-F) or the confinement radius (G-I). EGF-Cy3B (ligand-bound) data is from 3 independent experiments, and Fab-Cy3B (total EGFR) data is from 5 independent experiments. Each experiment involved detection and tracking of >500 EGFR objects. *, $p < 0.05$. Statistical analysis and p-values are indicated in Supplementary Table 1. Source data are provided as a Source Data file.

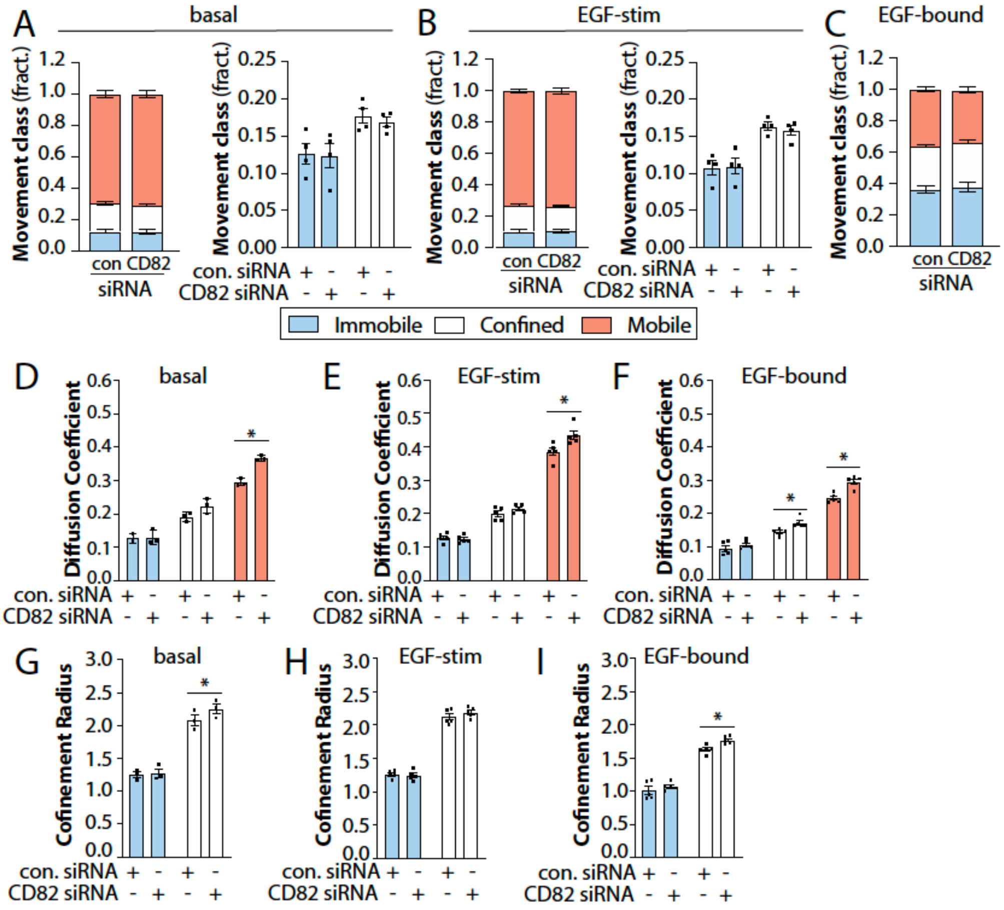

Supplementary Figure 6. Silencing CD82 impacts the diffusion coefficient of mobile EGFR. ARPE-19 cells were treated with siRNA to silence CD82 or non-targeting siRNA (control), as indicated. (A-I) Results of SPT analysis. The cells were then subjected to SPT using either Fab-Cy3B to label total EGFR in the absence (A, D, G) or presence (B, E, H) of unlabelled EGF, or labelled using EGF-Cy3B (C, F, I), to label only ligand-bound EGFR). Shown in A-C are the mean ± SE of the fraction of EGFR tracks in each mobility category (immobile, confined, mobile) under each condition, as well as in A-B the same data re-plotted to view the immobile and confined fractions. Also shown are mean ± SE of diffusion coefficient (D-F) or the confinement radius (G-I). EGF-Cy3B (ligand-bound) data is from 3 independent experiments, and Fab-Cy3B (total EGFR) data is from 5 independent experiments. Each experiment involved detection and tracking of >500 EGFR objects. *, $p < 0.05$. Statistical analysis and p-values are indicated in Supplementary Table 1. Source data are provided as a Source Data file.

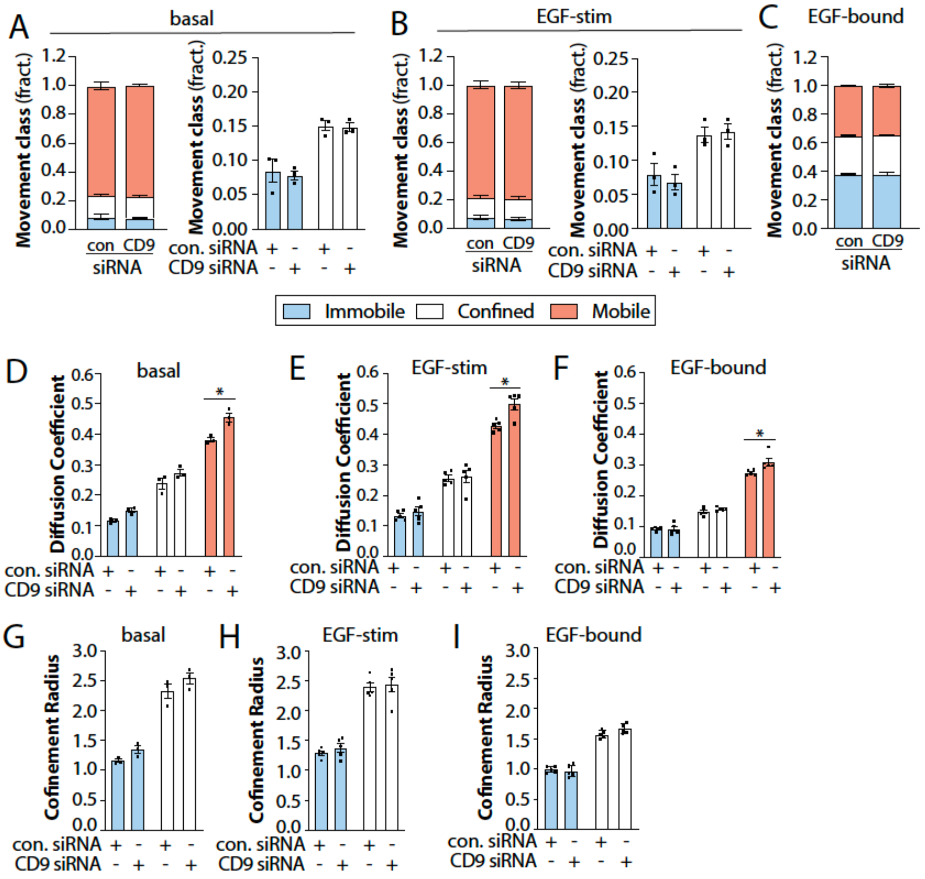

Supplementary Figure 7. Silencing CD9 impacts the diffusion coefficient of mobile EGFR. ARPE-19 cells were treated with siRNA to silence CD9 or non-targeting siRNA (control), as indicated. (A-I) Results of SPT analysis. The cells were then subjected to SPT using either Fab-Cy3B to label total EGFR in the absence (A, D, G) or presence (B, E, H) of unlabelled EGF, or labelled using EGF-Cy3B (C, F, I), to label only ligand-bound EGFR). Shown in A-C are the mean ± SE of the fraction of EGFR tracks in each mobility category (immobile, confined, mobile) under each condition, as well as in A-B the same data re-plotted to view the immobile and confined fractions. Also shown are mean ± SE of diffusion coefficient (D-F) or the confinement radius (G-I). EGF-Cy3B (ligand-bound) data is from 5 independent experiments, and Fab-Cy3B (total EGFR) data is from 3 independent experiments. Each experiment involved detection and tracking of >500 EGFR objects. *, $p < 0.05$. Statistical analysis and p-values are indicated in Supplementary Table 1. Source data are provided as a Source Data file.

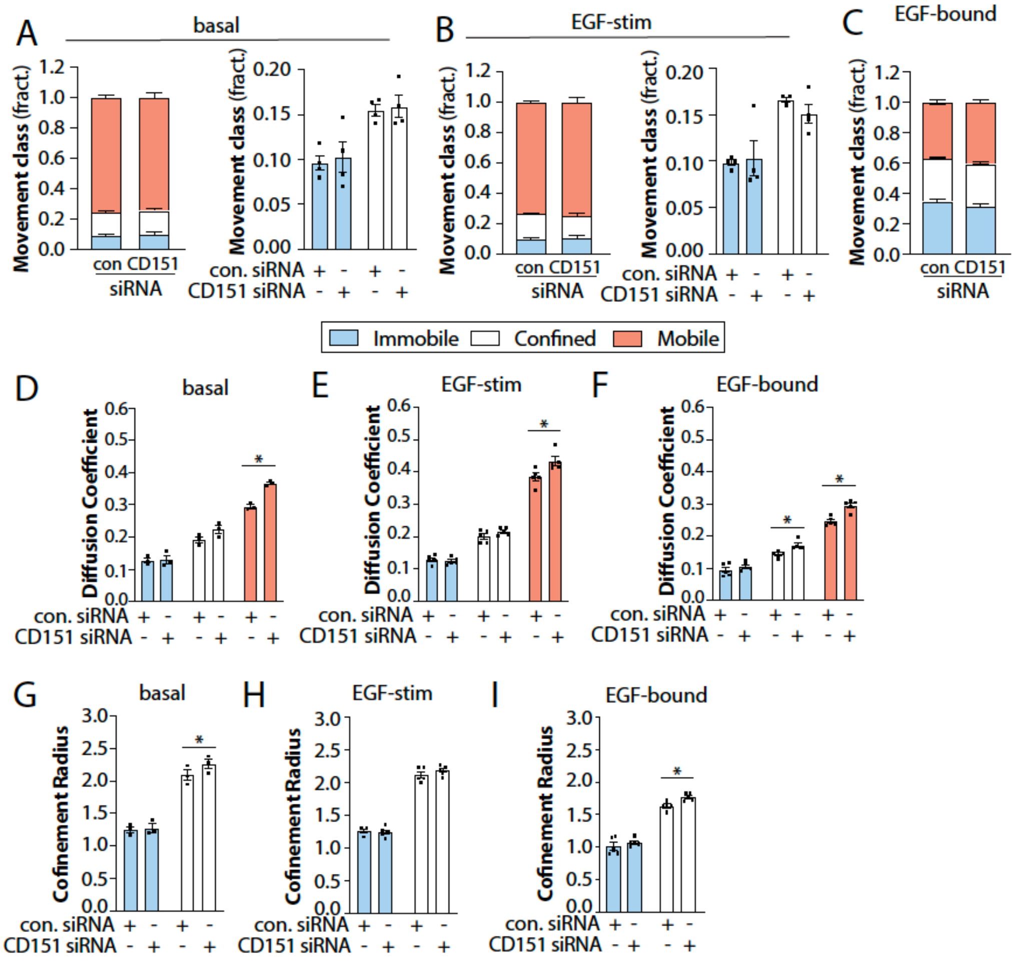

Supplementary Figure 8. Silencing CD151 impacts the diffusion coefficient of mobile EGFR. ARPE-19 cells were treated with siRNA to silence CD151 or non-targeting siRNA (control), as indicated. (A-I) Results of SPT analysis. The cells were then subjected to SPT using either Fab-Cy3B to label total EGFR in the absence (A, D, G) or presence (B, E, H) of unlabelled EGF, or labelled using EGF-Cy3B (C, F, I), to label only ligand-bound EGFR). Shown in A-C are the mean ± SE of the fraction of EGFR tracks in each mobility category (immobile, confined, mobile) under each condition, as well as in A-B the same data re-plotted to view the immobile and confined fractions. Also shown are mean ± SE of diffusion coefficient (D-F) or the confinement radius (G-I). EGF-Cy3B (ligand-bound) data is from 5 independent experiments, and Fab-Cy3B (total EGFR) data is from 3 independent experiments. Each experiment involved detection and tracking of >500 EGFR objects. *, $p < 0.05$. Statistical analysis and p-values are indicated in Supplementary Table 1. Source data are provided as a Source Data file.

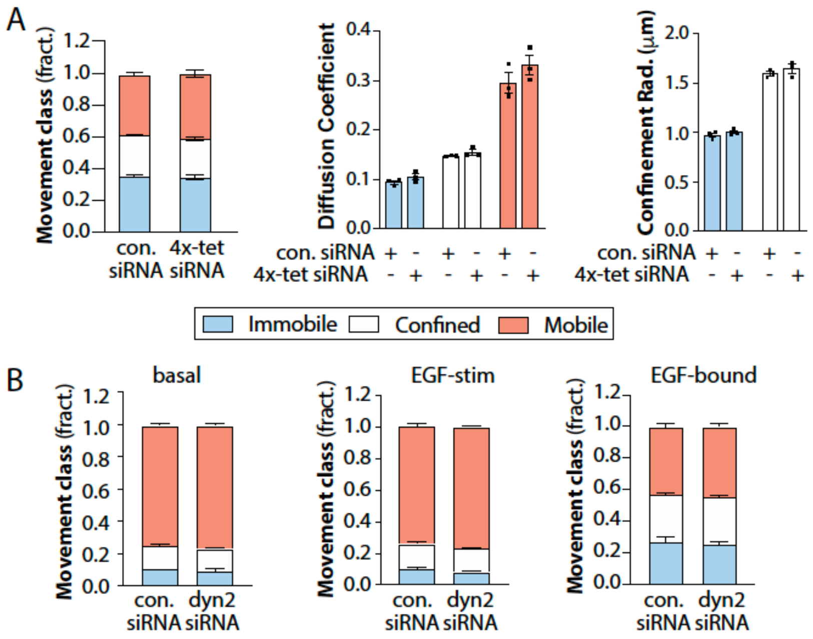

Supplementary Figure 9. Concomitant silencing of multiple tetraspanins does not alter mobility of ligand-bound EGFR. (A) ARPE-19 cells were treated with siRNA to silence individual tetraspanin proteins, or CD9, CD81, CD82 and CD151 concomitantly (4x-tet), or non-targeting siRNA (control). The cells were then subjected to SPT using EGF-Cy3B to label ligand-bound EGFR. Shown are the results of the SPT analysis, showing the fraction of EGFR tracks in each mobility category (immobile, confined, mobile) under each condition (left panel), diffusion coefficient of EGFR by mobility class (middle panel) and confinement radius of EGFR in immobile and confined EGFR populations (right panel). (B) APRE-19 cells were subject to treatment with siRNAs targeting dynamin2 (dyn2) or non-targeting siRNA (control, con.). The cells were then subjected to SPT using either Fab-Cy3B to label total EGFR in the absence (left panel) or presence (middle panel) of unlabelled EGF or labelled using EGF-Cy3B (right panel) to label only ligand-bound EGFR). Shown are the mean ± SE of the fraction of EGFR tracks in each mobility category (immobile, confined, mobile) under each condition. The data is from 3 independent experiments and each experiment involved detection and tracking of >500 EGFR objects. Statistical analysis and p-values are indicated in Supplementary Table 1. Source data are provided as a Source Data file.

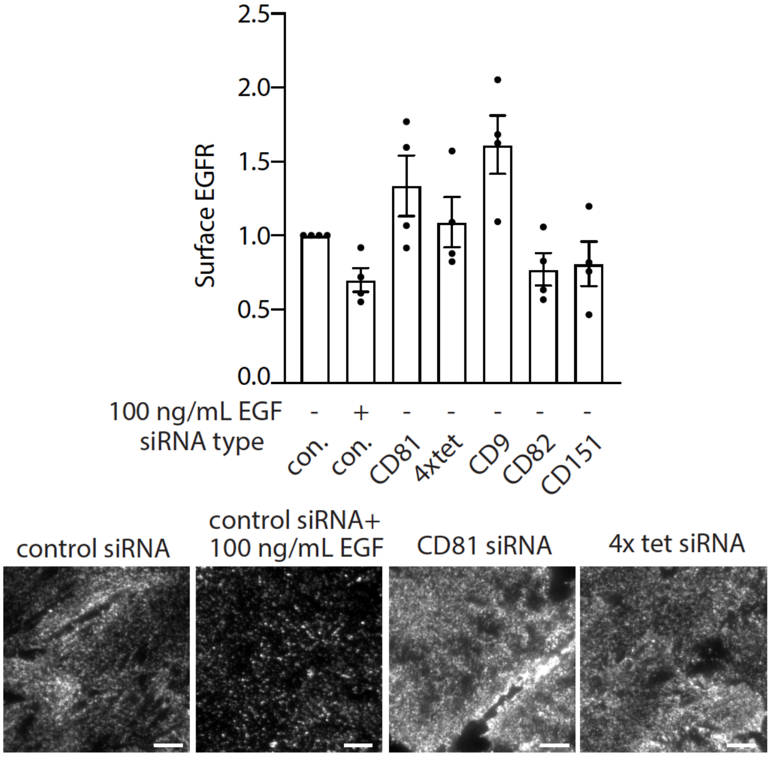

Supplementary Figure 10. Measurement of cell surface EGFR levels following tetraspanin silencing. ARPE-19 cells were transfected with siRNAs targeting tetraspanins as indicated. Following transfection, intact (non-permeabilized) cells were labelled with mAb108 (detecting surface-exposed EGFR), followed by fixation and labeling with appropriate secondary antibodies. Shown are representative microscopy images (bottom panels) as well as measurement of cell surface EGFR labeling intensity, showing the overall mean (bar) ± SE, as well as the mean from individual experiments (dots). Scale = 20 μm. The change in signal distribution and intensity upon treatment with 100 ng/mL EGF in control siRNA treated conditions supports the specificity of this antibody labeling. Source data are provided as a Source Data file.

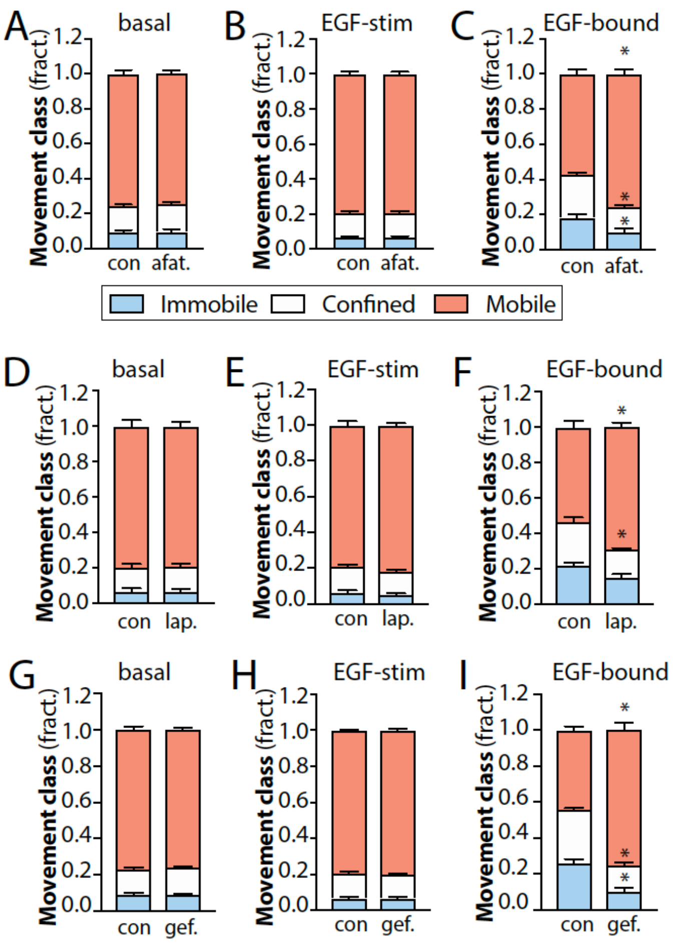

Supplementary Figure 11. EGFR tyrosine kinase inhibitors (TKIs) selectively impact confinement of ligand-bound EGFR. ARPE cells were treated with 2 µM of each TKI as indicated. The cells were then subjected to SPT using either Fab-Cy3B to label total EGFR in the absence (A, D, G) or presence (B, E, H) of unlabelled EGF, or labelled using EGF-Cy3B (C, F, I), to label only ligand-bound EGFR). Shown are the mean ± SE of the fraction of EGFR tracks in each mobility category (immobile, confined, mobile) under each condition. All data is from 3 independent experiments. Each experiment involved detection and tracking of >500 EGFR objects. *, *p*< 0.05. Statistical analysis and p-values are indicated in Supplementary Table 1. Source data are provided as a Source Data file.

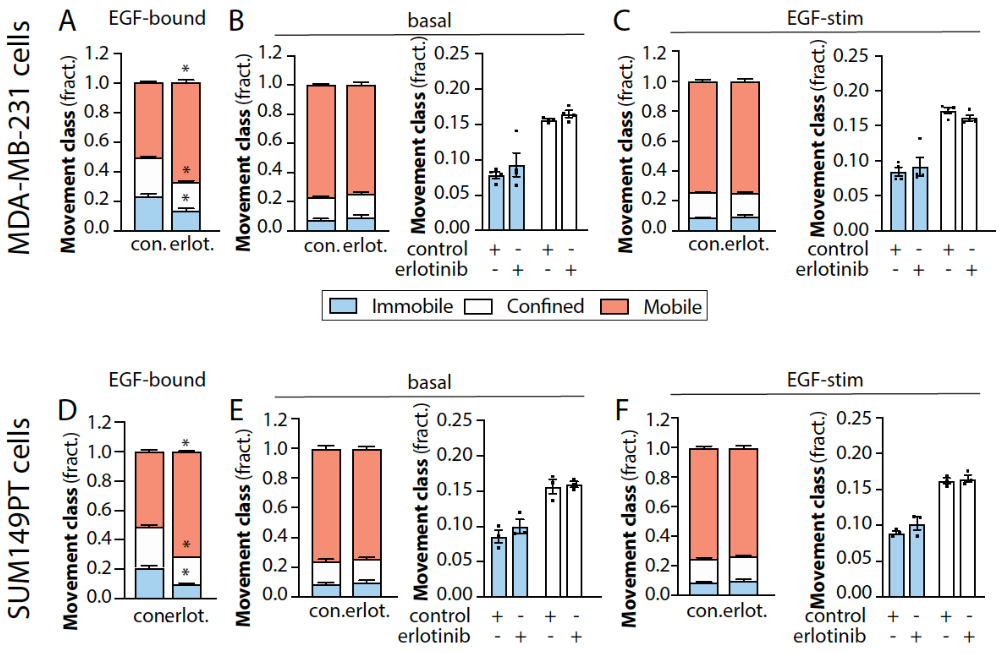

Supplementary Figure 12. Erlotinib selectively impacts mobility of ligand-bound EGFR in MDA-MB-231 and SUM149PT breast cancer cells. MDA-MB-231 cells (A-C) or SUM149-PT cells (D-F) were pre-treated with 2 μM erlotinib for 20 min. The cells were then subjected to SPT using either using EGF-Cy3B (A,B), to label only ligand-bound EGFR) or Fab-Cy3B to label total EGFR in the absence (B, E) or presence (C, F) of unlabelled EGF, or. Shown are the mean ± SE of the fraction of EGFR tracks in each mobility category (immobile, confined, mobile) under each condition, as well as in B-C, E-F the same data re-plotted to view the immobile and confined fractions. All data is from 3 independent experiments. Each experiment involved detection and tracking of >500 EGFR objects. *, $p < 0.05$. Statistical analysis and p-values are indicated in Supplementary Table 1. Source data are provided as a Source Data file.
