# Supplementary material for: Confinement of unliganded EGFR by tetraspanin nanodomains gates EGFR ligand binding and signaling
Source: Nat Commun. 2023 May 9;14:2681. doi: 10.1038/s41467-023-38390-z (PMC10170156; doi:10.1038/s41467-023-38390-z)
Supplement: Supplementary file 3 — Reporting Summary [file 41467_2023_38390_MOESM3_ESM.pdf]

nature portfolio

Corresponding author(s): Costin N. Anotnescu

Last updated by author(s): Apr 6, 2023

Reporting Summary

Nature Portfolio wishes to improve the reproducibility of the work that we publish. This form provides structure for consistency and transparency in reporting. For further information on Nature Portfolio policies, see our Editorial Policies and the Editorial Policy Checklist.

Statistics

For all statistical analyses, confirm that the following items are present in the figure legend, table legend, main text, or Methods section.

n/a	Confirmed	
☐	☒	The exact sample size (n) for each experimental group/condition, given as a discrete number and unit of measurement
☐	☒	A statement on whether measurements were taken from distinct samples or whether the same sample was measured repeatedly
☐	☒	The statistical test(s) used AND whether they are one- or two-sided<br>*Only common tests should be described solely by name; describe more complex techniques in the Methods section.*
☒	☐	A description of all covariates tested
☐	☒	A description of any assumptions or corrections, such as tests of normality and adjustment for multiple comparisons
☐	☒	A full description of the statistical parameters including central tendency (e.g. means) or other basic estimates (e.g. regression coefficient) AND variation (e.g. standard deviation) or associated estimates of uncertainty (e.g. confidence intervals)
☐	☒	For null hypothesis testing, the test statistic (e.g. F, t, r) with confidence intervals, effect sizes, degrees of freedom and P value noted<br>*Give P values as exact values whenever suitable.*
☒	☐	For Bayesian analysis, information on the choice of priors and Markov chain Monte Carlo settings
☒	☐	For hierarchical and complex designs, identification of the appropriate level for tests and full reporting of outcomes
☒	☐	Estimates of effect sizes (e.g. Cohen's d, Pearson's r), indicating how they were calculated

Our web collection on statistics for biologists contains articles on many of the points above.

Software and code

Policy information about availability of computer code

Data collection	Micrsocopy images were obtained as described in the Materials and Methods, using MetaMorph (Molecular Devices, San Jose, CA).
Data analysis	There is no new code or software developed specifically for this study.<br>The analysis of single particle tracking was done in Matlab using uTrack software, initially described in Jaqaman et al. Nature Methods, 2008, 5:695-702 available here: https://github.com/DanuserLab/u-track<br>The intensity-based co-localization analysis within nanodomains or EGFR objects detected in TIRF microscopy images was done in Matlab using the runDetection function, part of the cmeAnalysis pipeline, is as described in Aguet et al. 2013. Dev. Cell. 26:279-291, available here: https://github.com/DanuserLab/cmeAnalysis. Additional applications and validation of this method were as described in Cabral-Dias, Lucarelli, et al. J Cell Biol. 2022, 221:e201808181<br>The colocalization analysis not based on intensity was done in Matlab using novel 3-color conditional colocalization analysis algorithm (Vega-Lugo, da Rocha-Azevedo et al., J Cell Biol, 221:e202106129), available here: https://github.com/kjaqaman/conditionalColoc.<br>Data were imported into GraphPad Prism 9 software for statistical analysis and the presentation of data, with the exception of the data shown in Figure 2B-E and Figure 5K, for which the statistical analysis was performed in MATLAB (Mathworks, Natick, MA).

For manuscripts utilizing custom algorithms or software that are central to the research but not yet described in published literature, software must be made available to editors and reviewers. We strongly encourage code deposition in a community repository (e.g. GitHub). See the Nature Portfolio guidelines for submitting code & software for further information.

Data

Policy information about availability of data

All manuscripts must include a data availability statement. This statement should provide the following information, where applicable:

- Accession codes, unique identifiers, or web links for publicly available datasets
- A description of any restrictions on data availability
- For clinical datasets or third party data, please ensure that the statement adheres to our policy

The datasets generated during and/or analysed during the current study are available from the corresponding author on reasonable request. The amount of data represented by microscopy image files exceeds our capacity for providing access to this data in a public repository. Source data are provided with this paper.

Human research participants

Policy information about studies involving human research participants and Sex and Gender in Research.

Reporting on sex and gender	Experiments in this study were performed on cultured cell lines. The cell lines used were ARPE-19 (male), MDA-MB-231 (female) and SUM149-PT (female). We did not observe differences in key observations and measurements in these different cell lines. This includes examination of the effect of CD81 silencing on EGFR mobility, finding similar effects of CD81 silencing in MDA-MB-231 cells (Supplemental Figure 5) and ARPE-19 cells (Figure 3), as well as similar effects of treatment with the EGFR tyrosine kinase inhibitor erlotinib (Figure 8 and Supplemental Figure 12). We report the sex of each cell line in the Methods section.
Population characteristics	This study does not involve human research participants.
Recruitment	This study does not involve human research participants.
Ethics oversight	This study does not involve human research participants.

Note that full information on the approval of the study protocol must also be provided in the manuscript.

Field-specific reporting

Please select the one below that is the best fit for your research. If you are not sure, read the appropriate sections before making your selection.

☒ Life sciences ☐ Behavioural & social sciences ☐ Ecological, evolutionary & environmental sciences

For a reference copy of the document with all sections, see nature.com/documents/nr-reporting-summary-flat.pdf

Life sciences study design

All studies must disclose on these points even when the disclosure is negative.

Sample size	No statistical method was performed to calculate sample size. Sample sizes were determined based on standard practices in the field and were informed by variability of measurements within and across trial repeats. For most experiments, a minimum of three independent experiments were performed. For experiments that measure parameters in individual cells, at least 10 cells per experiment were subject to measurement. For transparency, both trial averages and individual measurements are presented where possible.
Data exclusions	No data was excluded in the study.
Replication	All experiments were replicated; a minimum of 3 independent experiments were conducted for each experiment presented in the manuscript. The number of replicates specified in the Methods and Individual Figure Legends, as well as in Supplemental Table 1.
Randomization	Experiments were performed on cultured cell populations which may have some genetic or phenotypic heterogeneity. Following seeding of >500,000 cells per condition/well, cell samples were randomly assigned for siRNA/drug treatment prior to live cell imaging or nanodomain analysis. Cells selected for microscopy imaging were from random fields (see below) using a secondary fluorescence channel, and at least 10 cells were imaged and analyzed per condition per experiment. Each experiment was independently performed at least 3 times. These measured allow for systematic sampling of cell populations, minimizing the potential convolution by any covariates. No further randomization was performed in the study.
Blinding	Investigators were not blinded to group allocation during data collection or analysis. Blinding was not utilized as investigators required knowledge of inherent variables (i.e. fluorophores, duration) and were demanding on available resources. For microscopy experiments involving measurement of EGFR/EGF mobility in live cells, random fields were identified using a secondary fluorescent channel (e.g. Hoechst 33342 or GFP-CLCa). Mobility analysis was performed by automated software on unedited TIRF microscopy time-lapse image series. Analysis of images from fixed-cell nanodomain experiments required cropping of TIRF stills to exclude regions devoid of cells; this was performed by randomly selecting regions using a secondary channel (i.e. non-EGFR/EGF channel). Notable is that the analysis of images is fully automated subsequent to image or image time-lapse acquisition, allowing systematic and unbiased analysis of receptor mobility and nanoscale

organization. Immunoblotting experiments were not subject to blinding, but loading controls and total protein controls ensured that sample uniformity for quantification purposes. No other blinding was performed.

Reporting for specific materials, systems and methods

We require information from authors about some types of materials, experimental systems and methods used in many studies. Here, indicate whether each material, system or method listed is relevant to your study. If you are not sure if a list item applies to your research, read the appropriate section before selecting a response.

Materials & experimental systems

n/a	Involved in the study
☐	☒ Antibodies
☐	☒ Eukaryotic cell lines
☒	☐ Palaeontology and archaeology
☒	☐ Animals and other organisms
☒	☐ Clinical data
☒	☐ Dual use research of concern

Methods

n/a	Involved in the study
☒	☐ ChIP-seq
☒	☐ Flow cytometry
☒	☐ MRI-based neuroimaging

Antibodies

Antibodies used	Antibodies used are described in the Materials and Methods, and reported in new Supplementary Table 2 and 3.
Validation	The sources of validation for each of the antibody used are as follows: CD81 (Supplemental Figure 2, this manuscript); CD82 (Supplemental Figure 2, this manuscript); CD151 (Supplemental Figure 2, this manuscript); Clathrin Heavy Chain (Cail et al. 2022, J Cell Biol, 221: e202109013); Caveolin-1 (Crewe C, et al. Cell. 2018. 175:695-708.e13); Flotillin-1 (Fekri et al., Scientific Reports 2019, 9:17768); EGFR pY1068 (manufacturer website); EGFR pY1173 (manufacturer website); EGFR pY845 (manufacturer website); EGFR pY992 (manufacturer website); EGFR sc-03-G (manufacturer website); EGFR mAb806 (reference 14, 66-68); EGFR mAb108 (cell surface immunostaining validated in Supplementary Figure 10); pAkt S473 (manufacturer website); Akt (manufacturer website); pPLCγ (manufacturer website); pERK (manufacturer website); ERK (manufacturer website); GAPDH (manufacturer website); Actin (manufacturer website).

Eukaryotic cell lines

Policy information about cell lines and Sex and Gender in Research

Cell line source(s)	MDA-MB-231, SUM149PT, and ARPE-19 cells were initially obtained from ATCC.
Authentication	None of the cell lines were authenticated.
Mycoplasma contamination	Mycoplasma testing is done by DAPI staining at least monthly, no mycoplasma has been detected by this method.
Commonly misidentified lines (See ICLAC register)	We have not used any cells lines listed on the ICLAC register, version 11
