# Supplementary figures and images for: Confinement of unliganded EGFR by tetraspanin nanodomains gates EGFR ligand binding and signaling
Source: Nat Commun. 2023 May 9;14:2681. doi: 10.1038/s41467-023-38390-z (PMC10170156; doi:10.1038/s41467-023-38390-z)

Flgure 7A

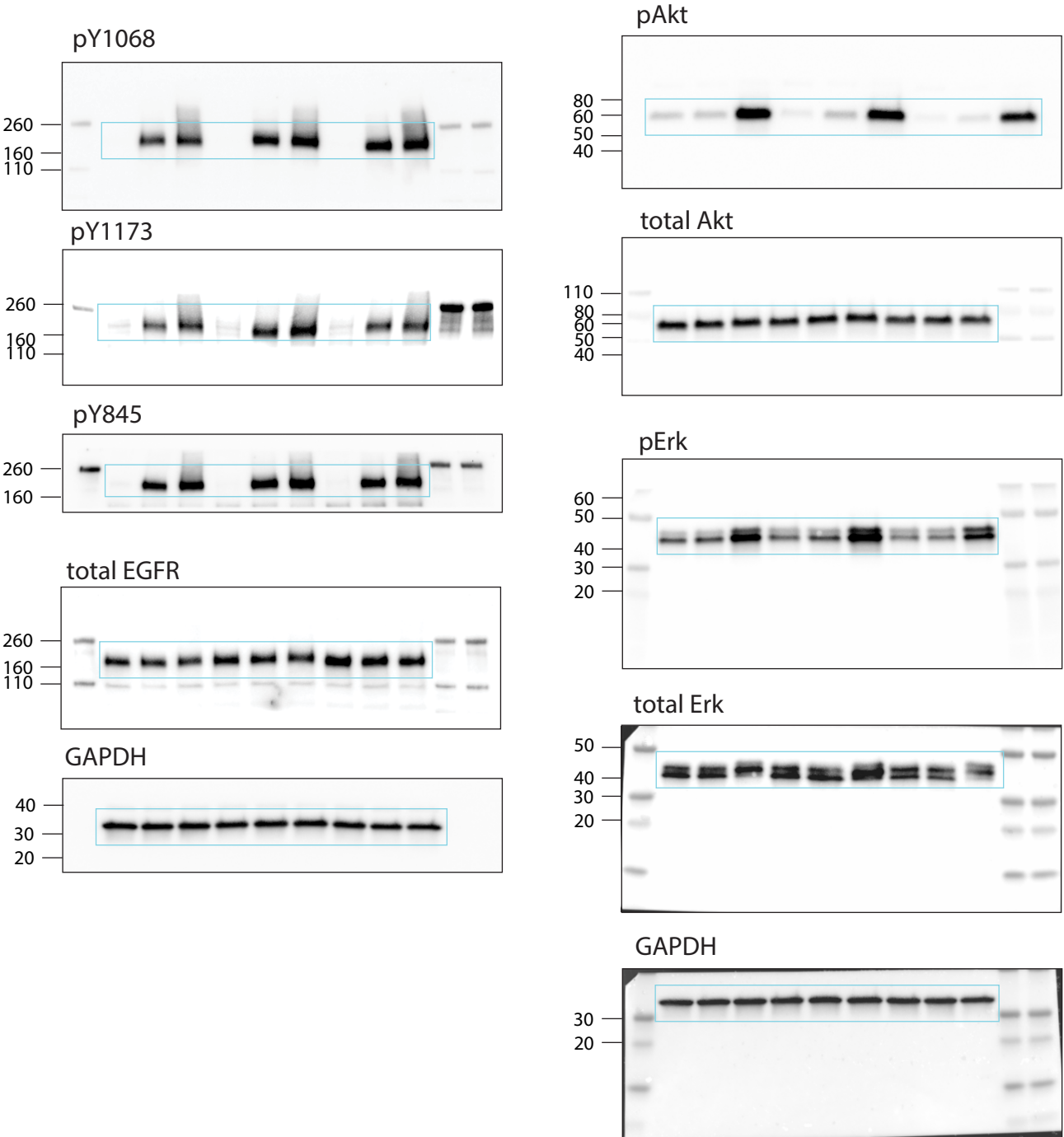

Figure 7F

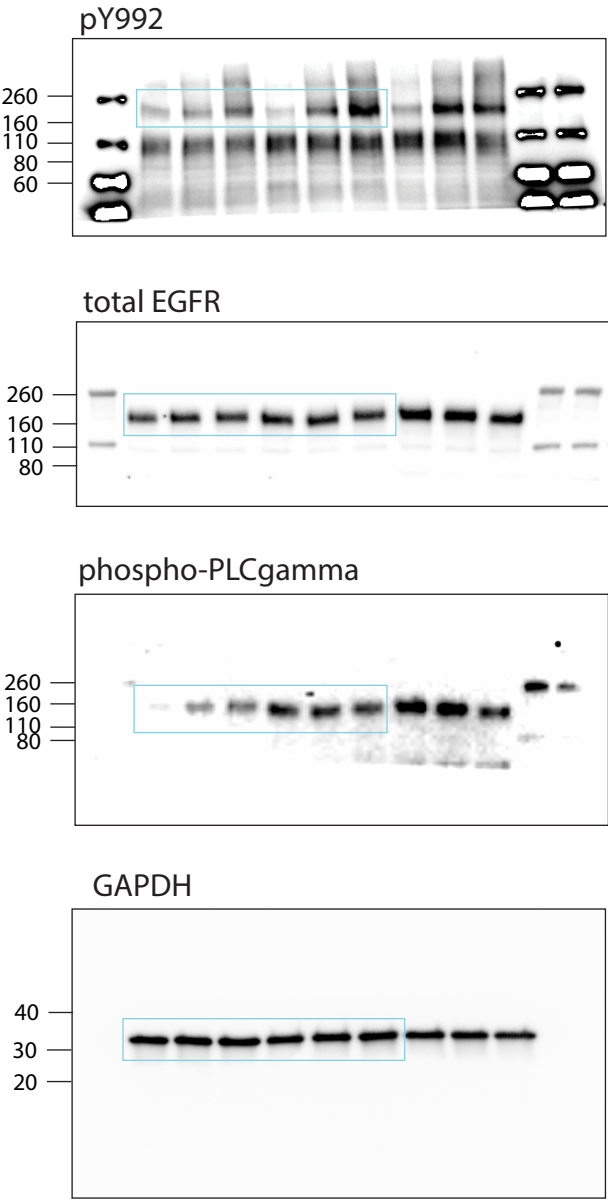

Figure S1B-C

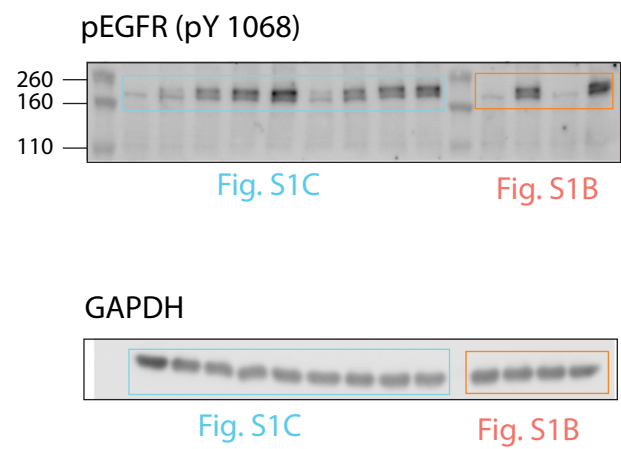

Figure S1F

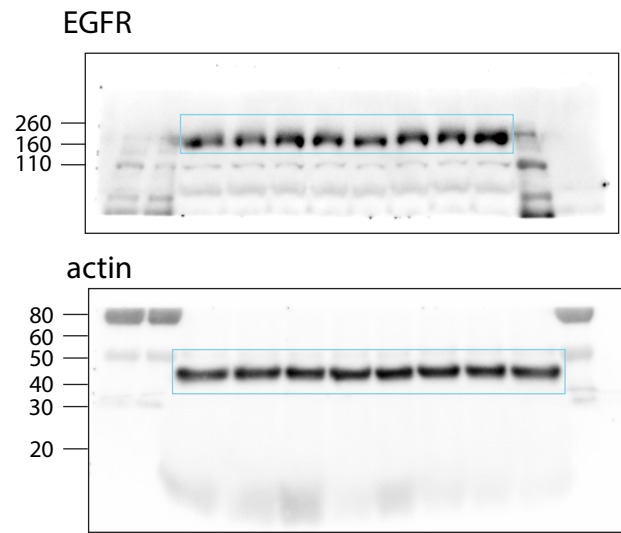

Supplement: Supplementary file 4 — Source Data [file 41467_2023_38390_MOESM4_ESM.zip › Antonsecu_Source Data.pdf]
